# Dual Involvement of Growth Arrest-Specific Gene 6 in the Early Phase of Human IgA Nephropathy

**DOI:** 10.1371/journal.pone.0066759

**Published:** 2013-06-24

**Authors:** Kojiro Nagai, Masashi Miyoshi, Takei Kake, Naoshi Fukushima, Motokazu Matsuura, Eriko Shibata, Satoshi Yamada, Kazuhiro Yoshikawa, Hiro-omi Kanayama, Tomoya Fukawa, Kunihisa Yamaguchi, Hirofumi Izaki, Akira Mima, Naoko Abe, Toshikazu Araoka, Taichi Murakami, Fumi Kishi, Seiji Kishi, Tatsuya Tominaga, Tatsumi Moriya, Hideharu Abe, Toshio Doi

**Affiliations:** 1 Department of Nephrology, Graduate School of Medicine, The University of Tokushima, Tokushima, Japan; 2 Chugai Research Institute for Medical Science, Inc., Shizuoka, Japan; 3 Department of Urology, Graduate School of Medicine, The University of Tokushima, Tokushima, Japan; 4 Department of Endocrinology, Diabetes and Metabolism, Kitasato University School of Medicine, Kanagawa, Japan; Fondazione IRCCS Ospedale Maggiore Policlinico & Fondazione D’Amico per la Ricerca sulle Malattie Renali, Italy

## Abstract

**Background:**

Gas6 is a growth factor that causes proliferation of mesangial cells in the development of glomerulonephritis. Gas6 can bind to three kinds of receptors; Axl, Dtk, and Mer. However, their expression and functions are not entirely clear in the different glomerular cell types. Meanwhile, representative cell cycle regulatory protein p27 has been reported to be expressed in podocytes in normal glomeruli with decreased expression in proliferating glomeruli, which inversely correlated with mesangial proliferation in human IgA nephropathy (IgAN).

**Methods:**

The aim of this study is to clarify Gas6 involvement in the progression of IgAN. Expression of Gas6/Axl/Dtk was examined in 31 biopsy proven IgAN cases. We compared the expression levels with histological severity or clinical data. Moreover, we investigated the expression of Gas6 and its receptors in cultured podocytes.

**Results:**

In 28 of 31 cases, Gas6 was upregulated mainly in podocytes. In the other 3 cases, Gas6 expression was induced in endothelial and mesangial cells, which was similar to animal nephritis models. Among 28 podocyte type cases, the expression level of Gas6 correlated with the mesangial hypercellularity score of IgAN Oxford classification and urine protein excretion. It also inversely correlated with p27 expression in glomeruli. As for the receptors, Axl was mainly expressed in endothelial and mesangial cells, while Dtk was expressed in podocytes. In vitro, Dtk was expressed in cultured murine podocytes, and the expression of p27 was decreased by Gas6 stimulation.

**Conclusions:**

Gas6 was uniquely upregulated in either endothelial/mesangial cells or podocytes in IgAN. The expression pattern can be used as a marker to classify IgAN. Gas6 has a possibility to be involved in not only mesangial proliferation via Axl, but also podocyte injury via Dtk in IgAN.

## Introduction

Gas6 is a growth factor which is post-translationally modified with γ- carboxylation of glutamate residues at its N terminus in the presence of vitamin K and inhibited by warfarin, an optional therapy for human kidney diseases. Gas6 was reported to be involved in the progression of glomerulonephritis and the development of diabetic nephropathy. Gas6 is expressed in the mesangial area in animal kidney disease models, such as rat anti-Thy-1 nephritis [1], anti-GBM nephritis [2] and streptozotocin induced diabetic rat and mouse model [3]. Gas6 induces proliferative and hypertrophic effects on mesangial cells, which lead to worsening of the kidney lesion. However, the expression of Gas6 in human kidney diseases is still unclear and yet to be fully examined.

Gas6 can bind to three kinds of receptors; Axl, Dtk (also called as Tyro3 or Sky), and Mer. Among them, Axl has the highest binding affinity with Gas6 and is expressed in endothelial and mesangial cells in animal kidney disease models [4], [5]. On the other hand, Dtk has an intermediate affinity and is expressed mainly in nerves and brain [6], [7]. However, a human glomerular SAGE transcriptome database revealed that Dtk does exist in glomeruli [8]. Previously, our group could not detect the expression of Dtk in cultured mouse mesangial cells [5]. As yet, to our knowledge, no one has examined what kind of cell type expresses Dtk in glomeruli.

Human IgA nephropathy (IgAN) is considered to be the most common form of glomerulonephritis in the world. However, the pathologic manifestations of IgAN are broad and can range from mild mesangial hypercellularity to a rapidly progressive glomerulonephritis with fulminant crescents and endocapillary proliferation. The outcome of IgAN varies greatly [9]. Therefore, it is possible that IgAN is a “syndrome” which can be divided into several subgroups according to etiology, histopathology, or clinical manifestation.

In order to predict the risk of progression of renal disease in IgAN, nephrologists use clinical information such as extent of proteinuria, presence of hypertension, and excretory renal function, which are consistently reported as prognostic factors [10]. Pathologists proposed the Oxford classification for the pathological classification of IgAN to define pathologic variables with acceptable inter-observer reproducibility [11]. Four of these variables: mesangial hypercellularity, segmental sclerosis, endocapillary hypercellularity, tubular atrophy/interstitial fibrosis were shown to have independent value in predicting renal outcome [12]. On one hand, basic researchers reported various molecules which are related with the progression of IgAN. Representative cell cycle regulatory protein p27 is one of the candidates, expressed mainly in podocytes in rat and human normal glomeruli. Expression levels of p27 are decreased in association with mesangial proliferation in experimental mesangial proliferative glomerulonephritis (rat anti-Thy-1 nephritis model) and human IgAN [13], [14].

In this study, we investigated whether expression of Gas6 and its receptors is involved in the progression of IgAN through analysis of the relationship between Gas6 expression and clinicopathological characteristics.

## Materials and Methods

### Ethics Statement

All clinical investigations were conducted according to the principles expressed in the Declaration of Helsinki and patients’ data were analyzed anonymously. All patients gave their informed, written consent. The study was approved by the Research Ethics Committee of Tokushima University or Kitasato University School of Medicine.

### Biopsies and Tissues

Forty patients with IgA-dominant immune deposits mainly in the mesangial area diagnosed in Tokushima University Hospital from June, 2010 to September, 2011 were enrolled. Among them, four cases with Henoch-Schönlein purpura nephritis, one case with diabetic nephropathy, three cases with nephrotic syndrome and one case with advanced sclerosing glomerulonephritis were excluded. As a result, 31 human renal biopsies were analyzed for this study. No patients enrolled were treated with warfarin. Six controls consisted of three biopsies from patients with asymptomatic hematuria and three 1-hour biopsies from living donor kidney transplants. They showed minor glomerular abnormality and negative immunofluorescence. The latter three transplant kidney biopsies were collected in Kitasato University hospital.

Human control kidney tissues for isolation of glomeruli were obtained in Tokushima University hospital from the uninvolved portion of tumor nephrectomy specimens, disclosing normal morphology and negative immunofluorescence. Control patient 1 was a 76 year old female, patient 2 a 76 yo male, and patient 3 an 81 yo male. Renal tissues were collected from only patients in whom serum creatinine levels were within normal limits, and in whom diabetic mellitus and proteinuria were absent. All samples were provided from Japanese.

### Demographic and Clinical Parameters

Demographics collected included age and gender at the time of biopsy. Clinical parameters collected included systolic and diastolic blood pressures, weight, height, serum creatinine, and 24-hour urine protein and urine protein-to-creatinine ratio within 1 month of the date of biopsy. GFR was estimated using the following new equation [15] applied for Japanese population: Estimated GFR (eGFR) (ml/min/1.73 m^2^) = 194×Serum Creatinine ^−1.094^×Age ^−0.287^ (If female×0.739).

### Pathological and Morphometric Analysis of Renal Biopsies

Biopsy adequacy was defined as a minimum of eight glomeruli available for examination by light microscopy. IgAN was confirmed as predominant IgA immunofluorescence in the mesangial area. Renal biopsy tissues were fixed in Dubosque-Brazil’s solution and stained with periodic acid-Schiff stain (PAS). Three independent nephrologists independently scored every feature according to the full Oxford score sheet [11], [12]. We derived an MEST score based on these results. Discordant scores were observed in a few cases and they were resolved by a meeting between the nephrologists. The mean values were applied to the analysis to see the association with the other variables.

### Immunohistochemical Analysis

The immunohistochemical analysis was performed on paraffin-embedded section using indirect immunohistochemistry procedure with the following primary antibodies: rabbit polyclonal antibody against Gas6 (Sigma-Aldrich, St. Louis, MO, USA), goat polyclonal antibody against Axl (R&D Systems, Minneapolis, MN, USA), nephrin (Santa Cruz Biotechnology, Dallas, TX, USA), and mouse monoclonal antibody against CD34 (Dako, Carpinteria, CA, USA) and p27 (BD, Franklin Lakes, NJ, USA), respectively. Frozen sections were stained to detect Dtk using indirect immunofluorescence method with mouse monoclonal antibody against Dtk (R&D Systems). Following the first antibody, sections were incubated with TSA™ Biotin System (PerkinElmer Life Sciences, Boston, MA, USA) and then with DAB (Wako Pure Chemical Industries, Osaka, Japan) or Texas Red Streptavidin (Vector Laboratories, Burlingame, CA, USA) for Gas6, Histofine SAB-PO(Goat) (Nichirei Biosciences, Tokyo, Japan) or biotin SP-conjugated donkey anti-goat antibody (Merck Millipore, Billerica, MA, USA) and then DAB or Texas Red Streptavidin for Axl, Alexa Fluor 488 or 594-conjugated donkey anti-goat antibody (Invitrogen, Grand Island, NY, USA) for nephrin, EnVision+ System- HRP Labelled Polymer (Dako) and then DAB for p27, Alexa Fluor 488-conjugated donkey anti-mouse antibody (Invitrogen) for CD34 and Dtk. The immunohistochemical signal of Gas6 was quantified using an image analyzer with a light microscope (Image Processor of Analytical Pathology; IPAP: Sumitomo Chemical Co., Tokyo, Japan) [3], [16]. Gas6 stained area was expressed as a percentage of total glomerular area occupied by Gas6 immunostained area. As for p27 positive cell number, p27 was endogenously expressed mainly in podocytes, but some minimal expression was observed in mesangial cells in normal glomeruli [14]. Therefore, three nephrologists who did not know each sample characteristics blindly examined the number without counting apparent mesangial positive cells. The result was divided by glomeruli number. The mean values of their data were shown. For each sample, at least eight glomerular profiles per patient were measured.

### Immunohistochemistry with Antigen Absorbed Primary Antibodies

No less than 10 molar excess of recombinant human Gas6, Axl-Fc or Dtk-Fc (R&D Systems) were mixed with the primary antibody, respectively, and rotated for 8 hours at 4°C. After centrifugation of the primary antibody only or pre-absorbed antibody by antigen at 3000 rpm for 10 min, sections were incubated with the supernatants, respectively. Human Fc portion (Bethyl Laboratories, Montgomery, TX, USA) was used for confirming the antigen specificity.

### Isolation of Glomeruli

The glomeruli isolated by sieving method were sonicated in cell extraction buffer (Mammalian cell extraction kit, Biovision Inc. Milpitas, CA, USA) and rotated for 1 hour at 4°C. After centrifugation of the samples, the supernatants were used as total lysates.

### Cell Culture Experiment

Conditionally immortalized murine podocytes were provided by Dr. Peter Mundel (Massachusetts General Hospital) [17]. Podocytes were maintained as previously described [18]. Podocytes between passage 20 to 25 were differentiated and stimulated with recombinant murine Gas6 (R&D Systems), or human TGFβ1 (PeproTech, Inc., Rocky Hill, NJ, USA) for 24 hours. Harvested cell lysates were suspended in cell extraction buffer and rotated for 1 hour at 4°C. After centrifugation of the samples, the supernatants were used as total cell lysates.

### Western Blotting

Lysates of glomeruli or harvested podocytes were applied to SDS-PAGE and immunoblotted with the primary antibodies indicated.

### Urine Gas6 Concentration

Urine samples were centrifuged at 3000rpm for 5 min. Supernatants stored at −80°C were rapidly thawed and centrifuged at 15000rpm for 15 min to remove any urates or phosphates before use in assays. Three times diluted samples were then applied on the ELIZA plate (human Gas6 Duoset ELIZA Development kit, R&D Systems). Data shown were the values collected by urine creatinine concentration.

### Statistical Analysis

All values are expressed as mean ± SD. Statistical analysis was performed using SPSS for Windows version 13.0 (SPSS, Inc., Chicago, IL, USA). Results were analyzed using paired t tests or Man-Whitney test. Correlation was evaluated by Spearman’s correlation coefficient by rank test. Significance was defined by *P* less than 0.05.

## Results

### IgAN was Divided into Two Subgroups According to Gas6 Expression Pattern

**Figure 1:**
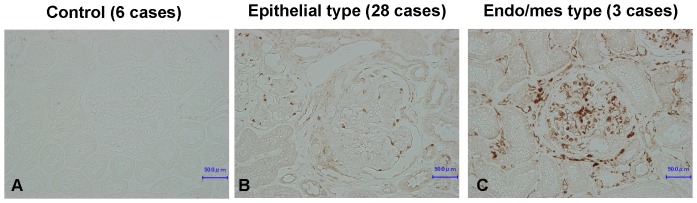
Gas6 expression in human IgA nephropathy. Biopsy samples were immunostained using indirect immunohistochemistry procedure with anti-Gas6 antibody. Representative images are shown in (A) from control patients, (B) from IgA nephropathy cases immunostained in epithelial cells, and (C) from IgA nephropathy cases immunostained in endothelial and mesangial cells. The original magnification was X200. Endo/mes, Endothelial and mesangial.

In order to examine the involvement of Gas6 in the progression of human IgAN, immunohistochemical analysis of Gas6 was performed in 6 controls and 31 IgAN patients enrolled. Then, unexpectedly, we found that Gas6 was expressed in podocytes (epithelial type) in 28 of 31 patients and in endothelial and mesangial cells (endo/mes type) in the other 3 cases (Figures 1, S1). We also confirmed the specificity of Gas6 staining by using antigen-absorbed primary antibody (Figure S2).

### Gas6 Expression Correlated with Clinical and Pathological Risk Factors

**Figure 2:**
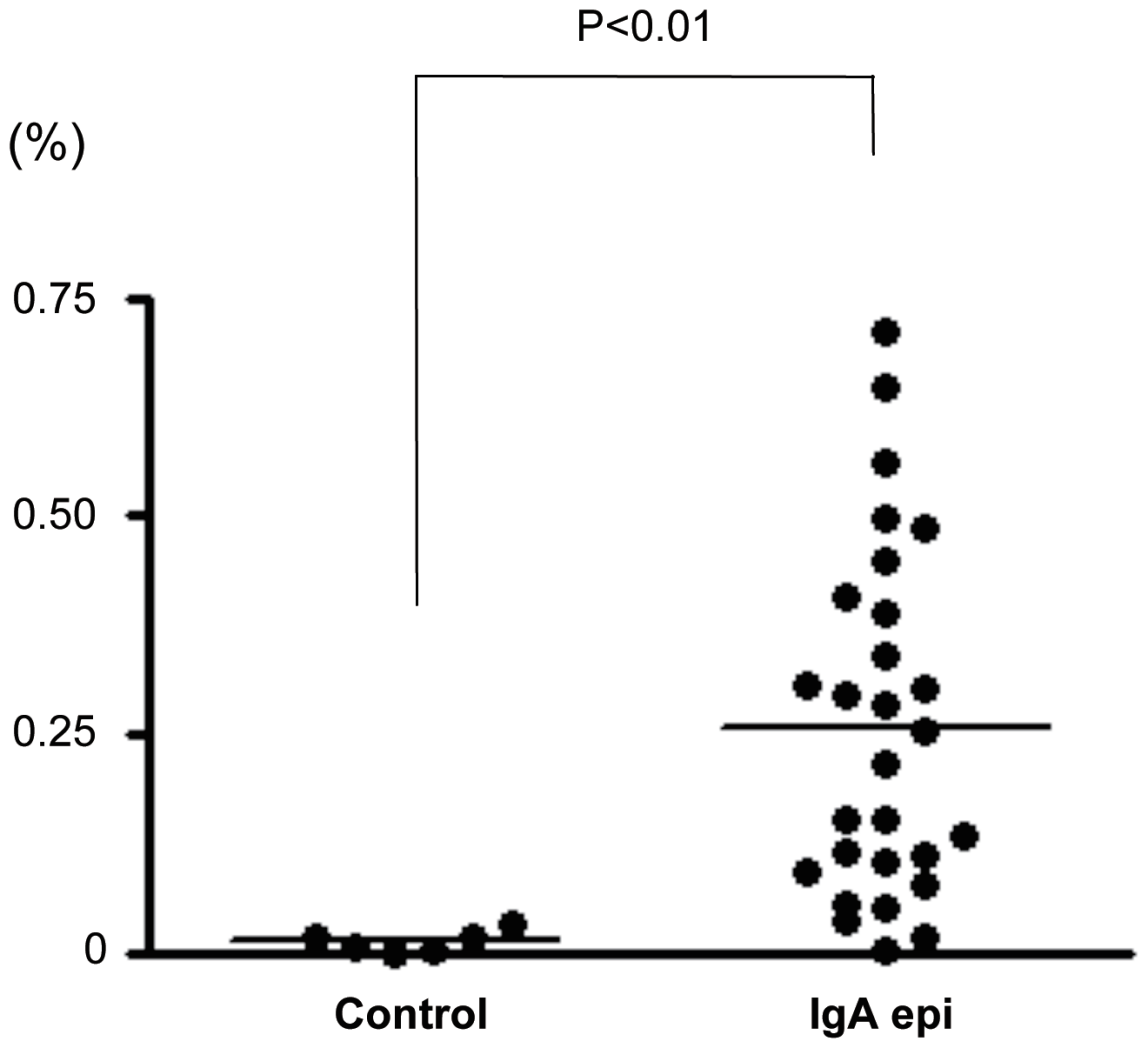
Gas6 stained area in human IgA nephropathy. Gas6 stained area is expressed as a percentage of total glomerular area occupied by Gas6 immunostained area. For each sample, at least eight glomerular profiles per patient were measured. IgA epi, IgA epithelial type.

**Figure 3:**
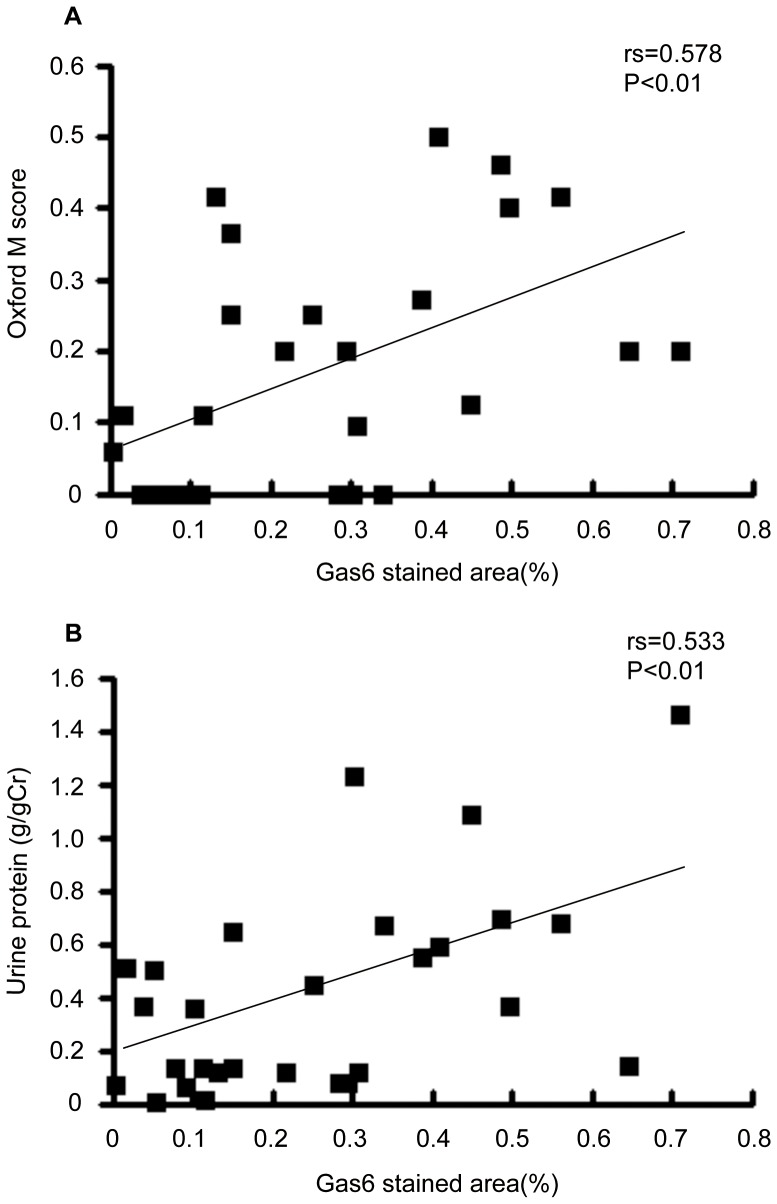
Correlation of Gas6 stained area with prognostic factors. Gas6 stained area in human IgA nephropathy correlated with (A) Oxford mesangial hypercellularity score and (B) urine protein excretion. N = 28. Oxford M score, Oxford mesangial hypercellularity score.

Next, we hypothesized that Gas6 is involved in the progression of human IgAN. Therefore, we compared the expression levels with risk factors for IgAN. Demographic, clinical parameters and pathological findings of controls and IgAN patients were shown in Table 1. Because only a few IgAN biopsies were endo/mes type, we could not compare the characterisitics of endo/mes type patients with those of controls or epithelial type patients. For that reason, we focused on epithelial type biopsies. Then, Gas6 stained area in human IgAN was significantly bigger than that in controls (Figure 2). Moreover, it correlated with a pathological risk factor, Oxford mesansigal hypercellularity score, and a clinical risk factor, urine protein excretion (Figure 3). On the other hand, it did not correlate with sex, age, presence of hypertension, Oxford segmental glomerulosclerosis, endocapillary hypercellularity, or tubular atrophy/interstitial fibrosis scores.

**Table 1:** Demographic, clinical parameters and pathological findings.

	Control (n = 6)	IgA epi (n = 28)	IgA e/m (n = 3)
Age	47.8±16.4	36.6±15.1	29.7±6.4
Women	4(66.7%)	14(50.0%)	2(66.7%)
eGFR(ml/min/1.73 m^2^ )	–	82.2±26.3	93.2±8.8
Urine protein(g/gCr)	–	0.405±0.381	0.371±0.188
Urine protein(g/day)	–	0.467±0.460	0.439±0.068
Oxford classification			
M score	–	0.165±0.167	0.321±0.197
S score 1	–	16(57.1%)	2(66.7%)
E score 1	–	6(21.4%)	3(100.0%)
T score 1 or 2	–	9(32.1%)	1(33.3%)
Hypertension	0(0.0%)	10(35.7%)	0(0.0%)

IgA epi, IgA epithelial type. IgA e/m, IgA endothelial and mesangial type.

M score, Mesangial hyprecellularity score. S score, Segmantal glomeruloscrelosis score. E score, Endocapillary hypercellularity score.

T score, Tubular atrophy/interstitial fibrosis score.

### Gas6 Expression Inversely Correlated with p27 Positive Cell Number

**Figure 4:**
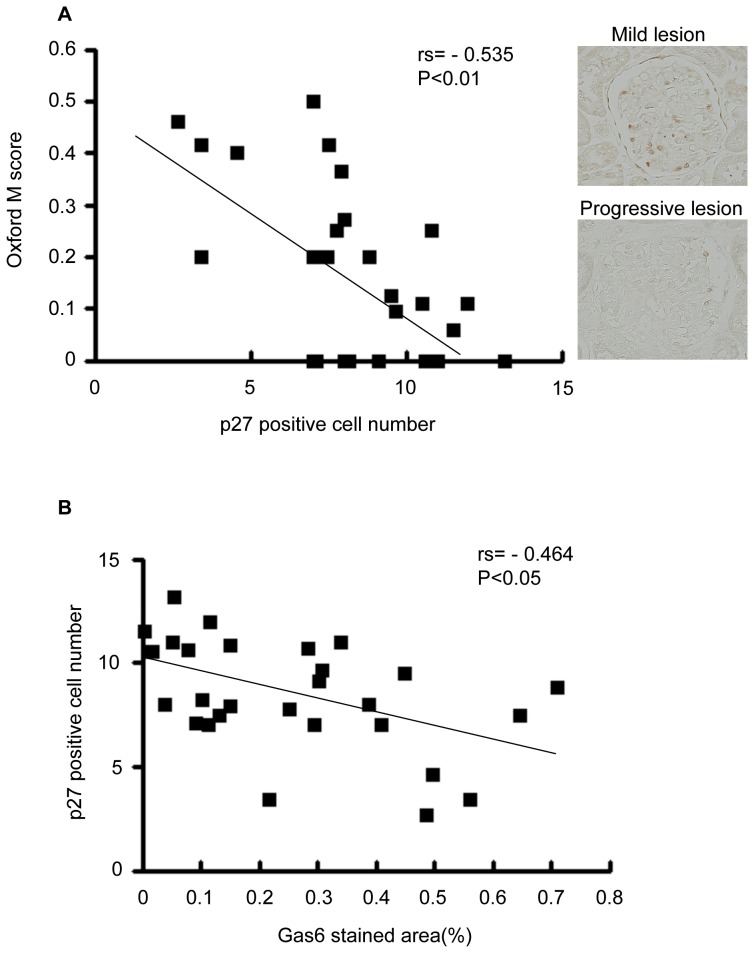
Inverse correlation of Gas6 stained area with p27 positive cell number. (A) Biopsy samples were immunostained using indirect immunohistochemistry procedure with anti-p27 antibody. p27 positive cell number was counted. The result was divided by glomeruli number. For each sample, at least eight glomerular profiles per patient were measured. p27 positive cell number correlated with Oxford mesangial hypercellularity score. (B) Gas6 stained area inversely correlated with p27 positive cell number. N = 28. Oxford M score, Oxford mesangial hypercellularity score.

To investigate the mechanism of Gas6 involvement in podocytes, we looked for the molecule which is expressed in podocytes and correlated with disease progression. One of the cell cycle regulatory proteins, p27 was reported to be expressed mainly in podocytes in normal glomeruli with decreased expression in proliferating glomeruli, which inversely correlated with mesangial proliferation in human IgAN [14]. Therefore, at first, we confirmed the relationship between p27 positive cell number and Oxford mesangial hypercellularity score (Figure 4A). Next, we found out that Gas6 stained area inversely correlated with p27 positive cell number (Figure 4B). This result suggests that Gas6 mediates progressive glomerular injury in human IgAN.

### Expression of Gas6 Receptors in Glomeruli

**Figure 5:**
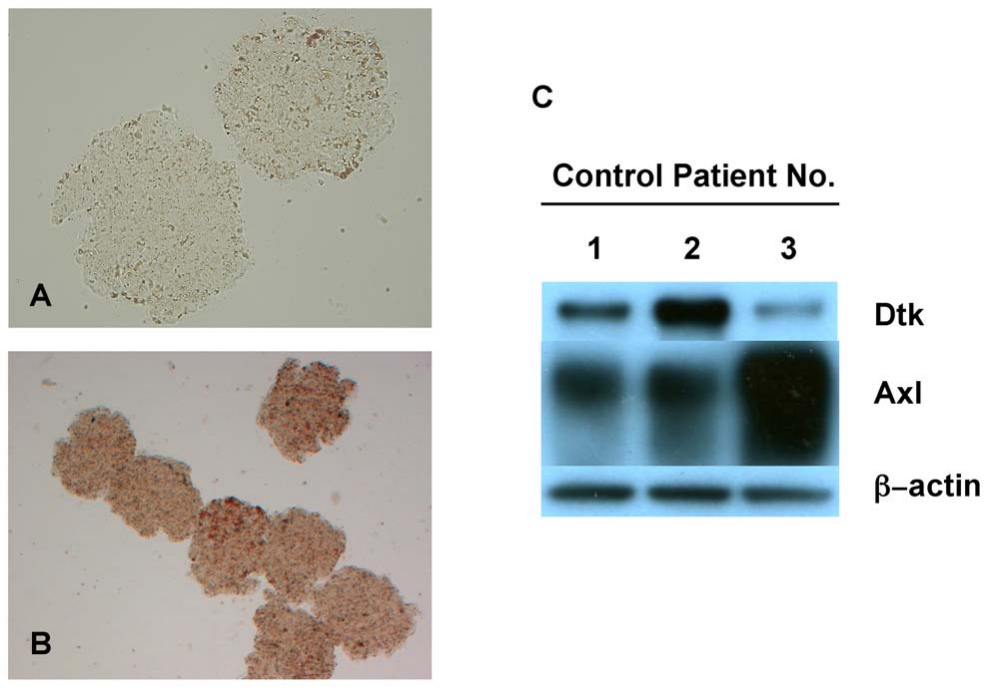
Expression of Dtk and Axl in control glomeruli. (A,B) Photographs of isolated glomeruli. The glomeruli were isolated from the uninvolved portion of tumor nephrectomy specimens by sieving method from three patients. The relative purity of isolated glomeruli was shown. The original magnification was (A) X100. (B) X50. (C) Ten µg of each glomerular lysate was analyzed by westernblotting with the antibodies indicated. Dtk and Axl were expressed in normal glomeruli.

**Figure 6:**
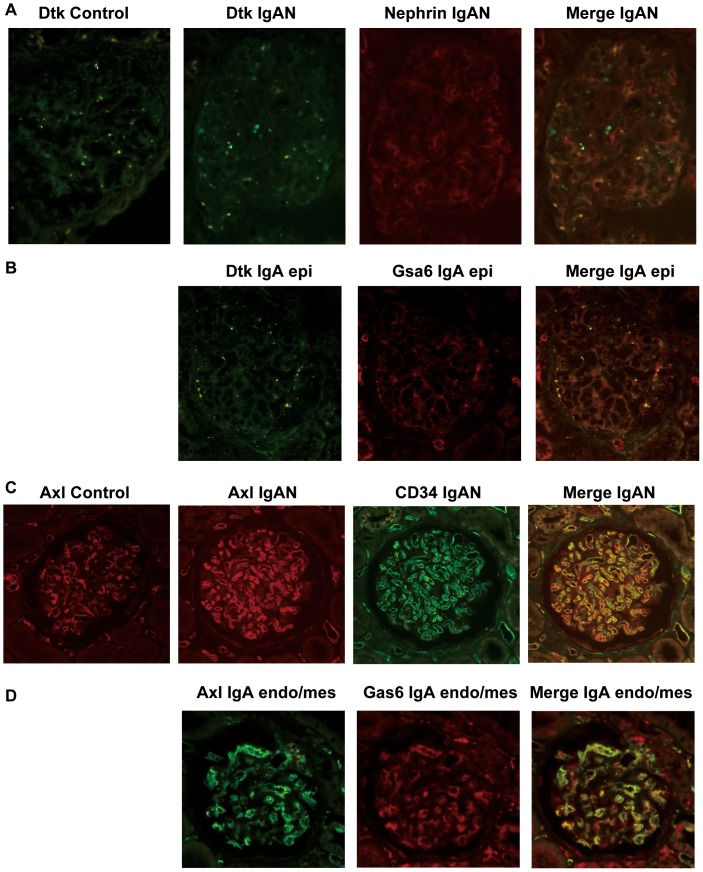
Double immunohistochemistry of Dtk and Axl with cell specific markers or Gas6. Biopsy samples were immunostained using indirect immunohistochemistry procedure with (A) anti-Dtk, anti-Nephrin (a podocyte marker), (B) anti-Dtk, anti-Gas6 or (C) anti-Axl, anti-CD34 (an endothelial cell marker), (D) anti-Axl, anti-Gas6 antibody. (A,B) Dtk immunostaining was detected in both control and IgA nephropathy, mostly merged with Nephrin. It was also merged with Gas6 in epithelial type IgA nephropathy. (C,D) Axl immunostaining was detected in both control and IgA nephropathy, mostly merged with CD34. It was also merged with Gas6 in endothelial and mesangial type IgA nephropathy. X200. IgAN, IgA nephropathy. IgA epi, IgA epithelial type. IgA endo/mes, IgA endothelial and mesangial type.

To further understand the role of Gas6 in podocytes and endothelial/mesangial cells, we investigated whether Gas6 receptors are expressed in normal glomeruli. Expression of Dtk and Axl was detected in human normal glomeruli lysates (Figure 5). Immunohistochemistrical analysis revealed that Dtk and Axl were expressed in podocytes and endothelial/mesangial cells, respectively, in normal and IgA glomeruli (Figures 6A,C, S3). Moreover, in IgAN, Gas6 was colocalized with Dtk/Axl (Figure 6B,D).

### Gas6 Downregulated p27 and Upregulated Dtk Expression in Podocytes in vitro

**Figure 7:**
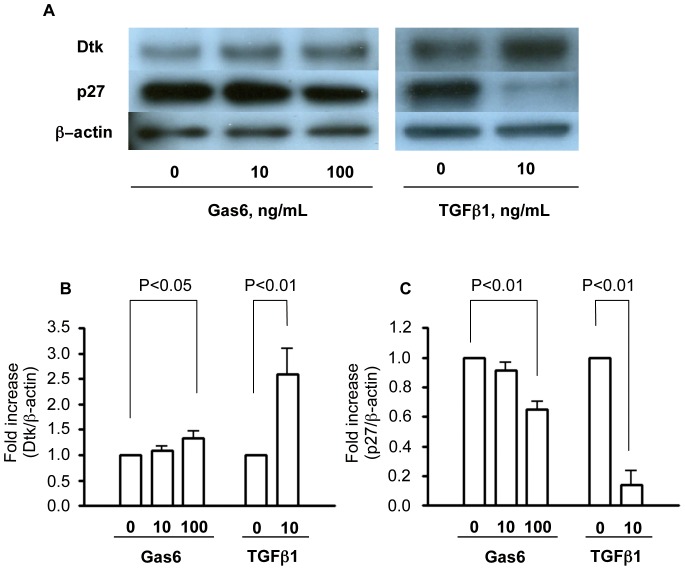
Effect of Gas6 on Dtk and p27 expression in podocytes. Podocytes were differentiated and stimulated with recombinant Gas6 or TGFβ1 for 24 hours. Cell lysates were subjected to immunoblotting with the antibodies indicated. Then, podocytes expressed Dtk. Gas6 could increase the expression of Dtk and reduce p27, as well as TGFβ1. Representative data were shown from four independent experiments. Quantitative examinations of the band density for (B) Dtk and (C) p27 were shown. The columns and error bars are the mean ± SD of four independent experiments.

To understand the function of Gas6 in podocytes, we stimulated murine cultured podocytes expressing Dtk with Gas6. Gas6 could reduce the expression of p27, as well as TGFβ1, the representative cytokine involved in the progression of IgAN (Figure 7A,C). These results are compatible with in vivo findings shown in Figure 4 and suggest that Gas6 is involved in podocyte injury. In addition, Gas6 also could increase Dtk receptor (Figure 7A,B).

## Discussion

Here we show the expression of Gas6 and its receptors in human IgAN. Gas6 was upregulated in either podocytes or endothelial/mesangial cells. In IgAN, Dtk was the receptor for Gas6 in podocytes, while Axl was in endothelial/mesangial cells. Gas6 expression in podocytes correlated with several prognostic factors, such as mesangial proliferation and urine protein excretion and was inversely associated with p27 expression. Gas6 was involved in human IgAN either via Dtk and Axl in podocytes and endothelial/mesangial cells, respectively.

Gas6 is a growth factor expressed in endothelial/mesangial cells with its receptor, Axl, which causes proliferation of mesangial cells in rat glomerulonephritis model [1]. However, in human IgAN, Gas6 upregulation was observed mainly in podocytes, while endothelial/mesangial dominant expression was seen in a few patients. The expression pattern can be a marker to classify IgAN, and we should re-evaluate the therapeutic effects and its prognosis according to the pattern.

In the cases with endo/mes type, according to a previous report [1], the role of Gas6 could be an exacerbation factor for glomerulonephritis through mesangial proliferation, and Axl was the receptor for Gas6. Though there is no statistic evidence, the pathology of endo/mes type tended to be more active than that of epithelial type because of high mesangial and endocapillary hypercellularity score (Table 1), which is consistent with the findings in rat anti-Thy-1 nephritis model which has endo/mes type Gas6 expression and shows an acute and fulminant mesangial proliferation. On the other hand, we have never determined the role of Gas6 in IgAN with epithelial type. As well as TGFβ1, Gas6 could be involved in podocyte injury by decreasing p27 expression, and Dtk was the receptor for Gas6. However, Gas6 was also reported to be a protective factor to prevent apoptosis in another cells [19], [20]. Because warfarin therapy is sometimes applied to IgAN patients, the role of Gas6/Dtk in podocytes should be clarified in the further study by finding and analyzing an animal kidney disease model in which Gas6/Dtk pathway is involved.

Dtk is expressed in brain and neuron cells [6], [7]. Dtk was also reported to be expressed in kidney abundantly [7], but no one has never determined the expression site in glomeruli. Unfortunately, we could not confirm Dtk receptor upregulation in IgAN patients, as well as the results in vitro (Figure 7A,B). Dtk immunostaining was only successful when we used frozen samples. Therefore, we could not quantify the Dtk staining intensity in enough number of glomeruli per patient. However, to our knowledge, this study is the first report to confirm the cell type in which Dtk is expressed and suggest the role of Dtk in kidney.

In previous reports, Mer mRNA is detected in peripheral blood mononuclear cells, bone marrow mononuclear cells and monocytes, but not in granulocytes. Despite the fact that Mer mRNA is expressed in neoplastic B and T cell lines, it is not detected in normal B or T lymphocytes. In normal human tissues, Mer mRNA is expressed at highest levels in ovary, prostate, lung and kidney [21], [22]. We tried to detect Mer protein in glomeruli by using two commercially available antibodies against human Mer. In normal human glomeruli, we could not detect consistent positive bands by westernblotting analysis. In kidney biopsy samples from control and IgAN patients, we could not get positive immunostaining in frozen samples and paraffin-embedded sections (data not shown). Therefore, we have not known the significance of Mer receptor in kidney, but it is still possible that Mer receptor plays some roles in human kidney diseases.

So far, many biomarkers related to IgAN histology have been reported. Concerning podocyte injury, urinary podocalyxin correlated with acute extracapillary abnormalities. The number of urinary podocytes was associated with glomerulosclerosis [23]. With regard to tubular damage, urinary kidney injury molecule-1 (KIM-1) and neutrophil gelatinase-associated lipocalin (NGAL) were markers for the detection of early tubular damage [24]–[26]. Relating to pathogensis and histolgical change of IgAN, the level of galactose-deficient IgA1 in the sera of patients with IgAN was associated with disease progression, but its relationship with histological severity was not analyzed [27]. The alternative complement pathway and the lectin pathway are involved in IgAN. Therefore, excretion of complement proteins, such as membrane attack complex, factor H, and mannose-binding lectin were associated with disease severity including acute and chronic lesions [28], [29]. Extracellular matrix is increased with the progression of IgAN. Therefore, the gene expression data of several proteoglycans from the tubulointerstitial compartment correlated with tubular atrophy/interstitial fibrosis [30]. Compared with these biomarkers, Gas6 expression was related to mesangial proliferation, but not to segmental glomerulosclerosis, endocapillary hypercellularity, or tubular atrophy/interstitial fibrosis. We assume that it is because Gas6 can be upregulated in the development of IgAN even without active lesions. It can be unique because most of the other proposed biomarkers were associated with acute or chronic histological injury. Therefore, urinary Gas6 concentration can be promising to detect the early phase of IgAN. We also investigated urine Gas6 concentration. Then, human IgA patients had higher Gas6 excretion than control (IgA epithelial type, IgA endo/mes type vs. Control. 1.01±1.06(N = 28), 2.15±1.95(N = 3) vs. 0.48±0.63(N = 32) µg/gCr, respectively). However, urine Gas6 concentrations of human IgA patients were not associated with prognostic markers such as the Oxford score or proteinuria (data not shown). We postulate that this is due to the relatively low sensitivity of commercially available ELIZA system. The establishment of highly sensitive Gas6 ELIZA system can be one of the promising methods to differentiate between nephritis and the other disease, such as bladder infection or urine stone.

Limitation of this study should be noted. First, this is a single-institution study. Second, most of the cases analyzed were in the early phase of IgAN with low proteinuria. It is partly because an established health check-up system in Japan. If we check advanced IgAN samples, more cases with endo/mes type might be found.

In summary, we could reveal the expression of Gas6 and its receptors, Axl and Dtk in IgAN. Surprisingly, in most patients, Gas6 was expressed in podocytes and Dtk was the receptor for Gas6. Human IgAN can be classified in two different types according to Gas6 expression pattern. We should review the prognosis of IgAN and effect of warfarin by dividing IgAN according to Gas6 expression pattern.

## Supporting Information

Figure S1 **Double immunohistochemistry of Gas6 with cell specific markers.** Biopsy samples were immunostained using indirect immunohistochemistry procedure with (A) anti-Gas6, anti-Nephrin (a podocyte marker) or (B) anti-Gas6, anti-CD34 (an endothelial cell marker) antibody. (A) Gas6 immunostaining was observed outside Nephrin in epithelial type IgA nephropathy. (B) Gas6 immunostaining mostly merged with CD34 in endothelial and mesangial type IgA nephropathy. X200. IgA epi, IgA epithelial type. IgA endo/mes, IgA endothelial and mesangial type.
(TIFF)

Figure S2 **Specificity of Gas6 immunohistochemistry.** Biopsy samples were immunostained using indirect immunohistochemistry procedure with (A) normal rabbit IgG, (B) anti-Gas6 antibody, and (C) antigen pre-absorbed anti-Gas6 antibody. The staining disappeared by antigen absorption almost completely. X100.
(TIFF)

Figure S3 **Specificity of Dtk and Axl immunohistochemistry.** Biopsy samples were immunostained using indirect immunohistochemistry procedure with (A) anti-Dtk antibody (X200) and (B) anti-Axl antibody (X100). The staining disappeared by Fc portion conjugated receptor protein absorption almost completely, but not by Fc portion only.
(TIF)
