# Supplementary figures and images for: Dual Involvement of Growth Arrest-Specific Gene 6 in the Early Phase of Human IgA Nephropathy
Source: PLoS One. 2013 Jun 24;8(6):e66759. doi: 10.1371/journal.pone.0066759 (PMC3691258; doi:10.1371/journal.pone.0066759)

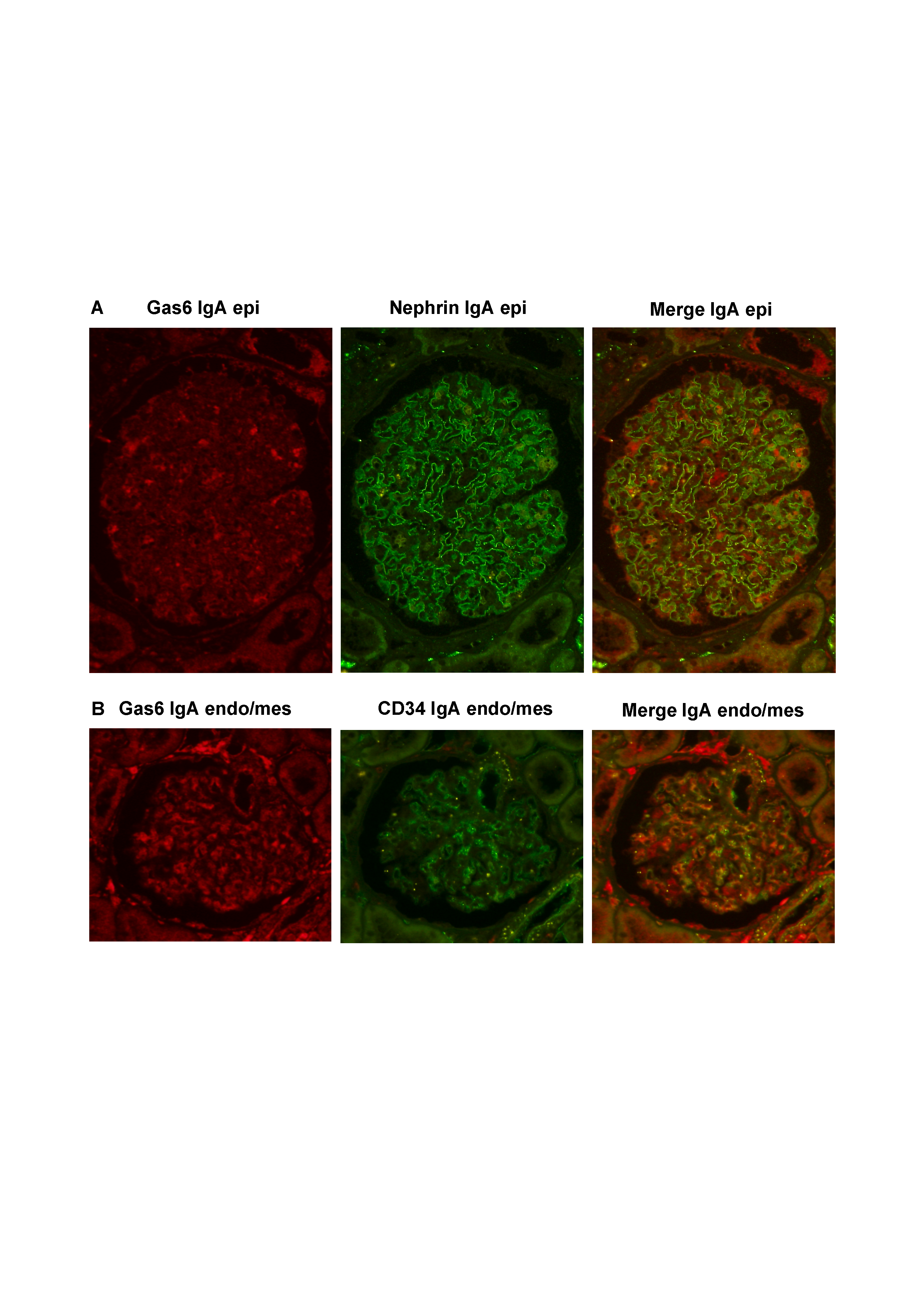

Supplement: Figure S1 — Double immunohistochemistry of Gas6 with cell specific markers. Biopsy samples were immunostained using indirect immunohistochemistry procedure with (A) anti-Gas6, anti-Nephrin (a podocyte marker) or (B) anti-Gas6, anti-CD34 (an endothelial cell marker) antibody. (A) Gas6 immunostaining was observed outside Nephrin in epithelial type IgA nephropathy. (B) Gas6 immunostaining mostly merged with CD34 in endothelial and mesangial type IgA nephropathy. X200. IgA epi, IgA epithelial type. IgA endo/mes, IgA endothelial and mesangial type. (TIFF) [file pone.0066759.s001.tiff]

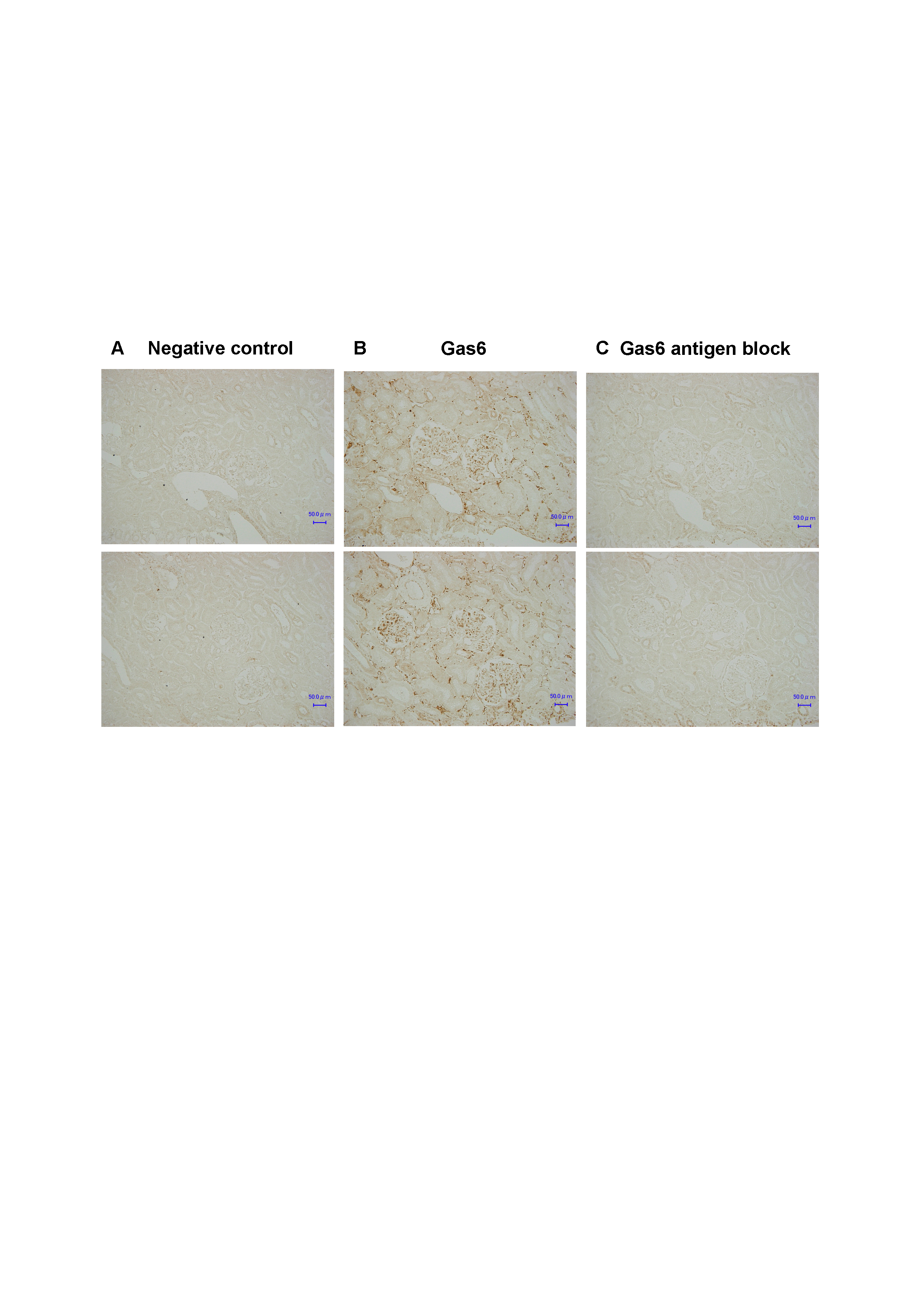

Supplement: Figure S2 — Specificity of Gas6 immunohistochemistry. Biopsy samples were immunostained using indirect immunohistochemistry procedure with (A) normal rabbit IgG, (B) anti-Gas6 antibody, and (C) antigen pre-absorbed anti-Gas6 antibody. The staining disappeared by antigen absorption almost completely. X100. (TIFF) [file pone.0066759.s002.tiff]

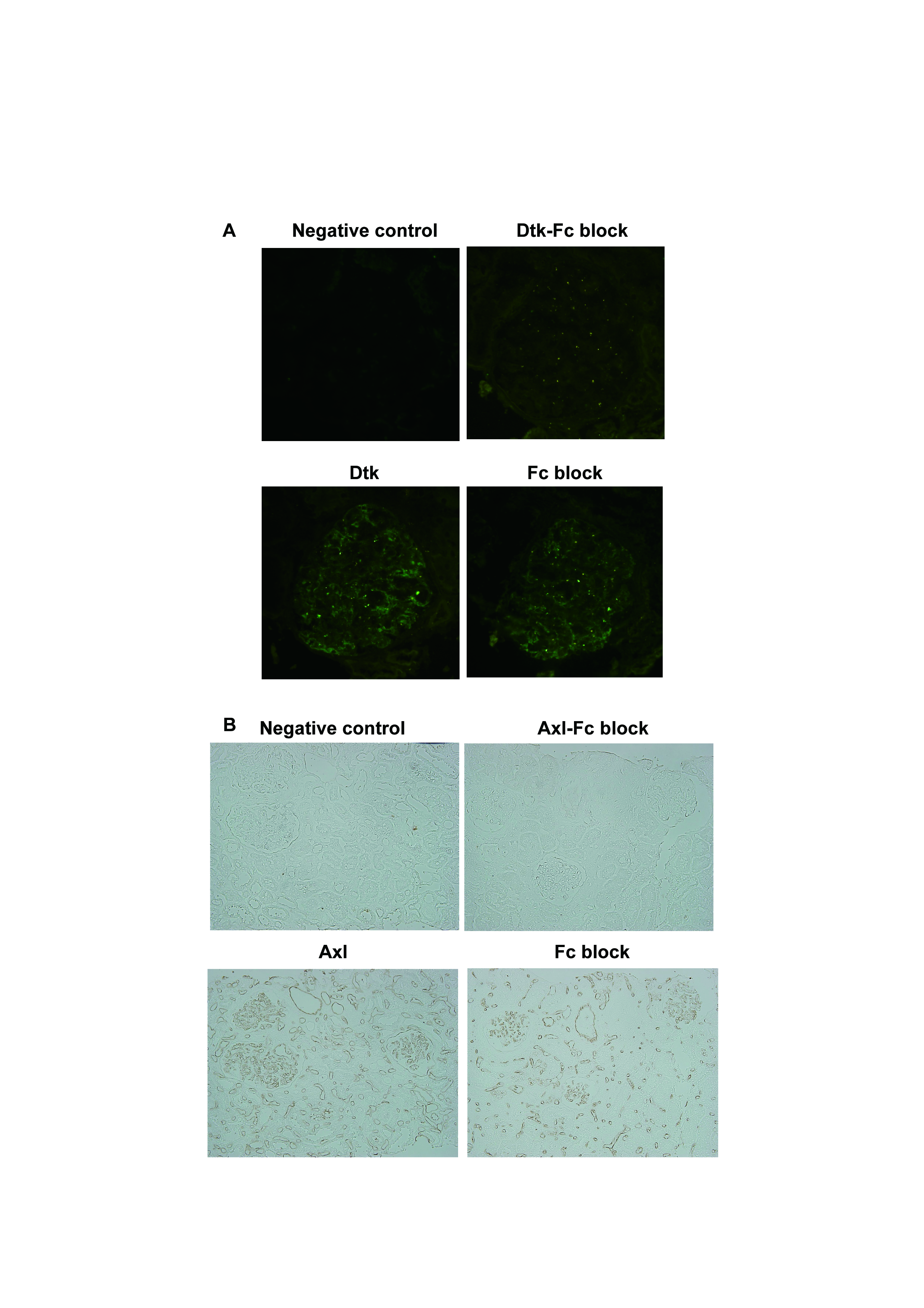

Supplement: Figure S3 — Specificity of Dtk and Axl immunohistochemistry. Biopsy samples were immunostained using indirect immunohistochemistry procedure with (A) anti-Dtk antibody (X200) and (B) anti-Axl antibody (X100). The staining disappeared by Fc portion conjugated receptor protein absorption almost completely, but not by Fc portion only. (TIF) [file pone.0066759.s003.tif]
